# Functional Complexity of Thermogenic Adipose Tissue: From Thermogenesis to Metabolic and Fibroinflammatory Crosstalk

**DOI:** 10.3390/ijms26189045

**Published:** 2025-09-17

**Authors:** Wael Jalloul, Irena Cristina Grierosu, Despina Jalloul, Cipriana Stefanescu, Vlad Ghizdovat

**Affiliations:** Department of Biophysics and Medical Physics-Nuclear Medicine, Grigore T. Popa University of Medicine and Pharmacy Iasi, 700115 Iasi, Romania

**Keywords:** brown adipose tissue, thermogenesis, beige adipocytes, batokines, insulin sensitivity, UCP1-independent pathways, metabolic syndrome

## Abstract

Brown adipose tissue (BAT) has shifted from being considered a transient thermogenic organ of infancy to a metabolically dynamic and multifunctional tissue throughout life. Histologically and developmentally distinct from white and beige adipocytes, BAT originates from a myogenic lineage and is characterised by a high mitochondrial density, multilocular lipid droplets, and abundant sympathetic innervation. Its defining function, non-shivering thermogenesis, is mediated by uncoupling protein 1 (UCP1) and complemented by alternative mechanisms such as futile creatine and calcium cycling. Beyond heat production, thermogenic fat is crucial in regulating whole-body metabolism. It contributes to glucose, lipid, and branched-chain amino acid homeostasis, and engages in endocrine and paracrine signalling through a rich secretome of batokines, lipid mediators, and extracellular vesicle-bound microRNAs. These signals orchestrate crosstalk with the liver, skeletal muscle, pancreas, and immune system, enhancing insulin sensitivity, vascularisation, and anti-inflammatory responses. Brown/Beige fat also exhibits notable anti-fibrotic properties and supports adipose tissue remodelling, maintaining structural and functional plasticity under metabolic stress. This review offers a comprehensive synthesis of thermogenic adipose tissue biology, integrating its structural, developmental, and molecular features with its expanding physiological functions, highlighting its pivotal role in energy balance as well as its emerging therapeutic potential in obesity, type 2 diabetes, and related metabolic disorders.

## 1. Adipose Tissue: Origins and Structural Characteristics

For a considerable period, adipose tissue was viewed primarily as an inert energy reservoir and, consequently, received limited scientific attention. This perception was further reinforced by societal biases, particularly among individuals attempting to lose weight, who associated fat accumulation with negative health outcomes. However, scientific advancements have profoundly reshaped this view, showing that adipose tissue plays complex roles in systemic physiology, including endocrine, immunomodulatory, and thermogenic functions [1].

From a cellular perspective, adipose tissue is far more heterogeneous than initially believed. While mature adipocytes account for the majority of tissue volume, particularly in white adipose depots, these cells represent only around 50% of the total cell population. The remaining fraction, known as the stromal vascular compartment, comprises preadipocytes, immune cells, endothelial cells, pericytes, nerve fibres, and fibroblasts. This diversity underlines the dynamic nature of adipose tissue and its ability to respond to varying physiological states [2,3].

Far from being solely structural, the extracellular matrix (ECM) of adipose tissue plays a central role in modulating cellular behaviour and facilitating tissue expansion. Composed of proteins like collagen (types I, III, and VI), laminin, fibronectin, and various proteoglycans, the ECM undergoes constant remodelling. These changes influence adipocyte differentiation, immune cell infiltration, vascularisation, and metabolic regulation. Dysregulation of ECM dynamics is closely associated with adipose dysfunction in metabolic disorders such as obesity and type 2 diabetes (T2D) [2,4,5].

In addition to adipocytes, adipose tissue exhibits numerous immune cells, including macrophages, lymphocytes, and mast cells, as well as extensive vascular and neural networks. The number and type of immune cells present vary depending on the tissue’s metabolic condition. For example, pro-inflammatory macrophages accumulate in obese states and contribute to insulin resistance, whereas anti-inflammatory phenotypes are more prevalent in lean tissue. This immunological environment is vital for maintaining local and systemic metabolic homeostasis [3,6].

Moreover, adipose tissue shows a notable capacity for regeneration. It is now well established that human fat cells are renewed at a steady rate of approximately 10% per year. This rate implies a complete turnover of adipocytes approximately every 10 to 12 years. Such regenerative dynamics are comparable to bone remodelling and highlight the tissue’s continuous adaptation to metabolic demands throughout life [7,8].

Considering the distinctions in origins, structure, growth, and function, professionals categorised adipose tissue into three principal varieties: white adipose tissue (WAT), brown adipose tissue (BAT), and beige adipose tissue (BeigeAT). WAT primarily stores energy, BAT dissipates energy through thermogenesis, and BeigeAT emerges in white depots under certain stimuli, exhibiting thermogenic properties. Each type differs in cellular origin, morphology, and metabolic role, justifying this classification within the broader adipose lineage [1,9]. While this review primarily addresses BAT, BeigeAT is also considered in contexts where its functions and regulatory mechanisms overlap or diverge from BAT, particularly in UCP1-independent thermogenesis.

### 1.1. White Adipose Tissue

The development of human WAT originates during mid-gestation, particularly between weeks 13 and 27 of foetal development, when progenitor cells derived from the mesoderm begin differentiating into preadipocytes. These cells ultimately mature into white adipocytes under the influence of local and systemic signalling factors, including hormonal, nutritional, and transcriptional regulators [1,10,11]. At birth, humans already possess structurally mature adipose depots in both subcutaneous and visceral regions, which expand in volume and distribution throughout infancy and adolescence [1].

White fat becomes the predominant form of adipose tissue in adulthood, typically representing 15–25% of total body mass in men and 25–35% in women, depending on age, sex hormones, and lifestyle factors [12,13]. Importantly, values above these ranges (≥35–40%) are generally associated with overweight or obesity rather than lean, healthy individuals. For the same body mass index, women generally exhibit about 10% higher fat mass than men, reflecting a fundamental sex difference in WAT distribution [14]. This adipose tissue deposition occurs primarily in the abdominal, gluteofemoral, and subcutaneous regions, although some variation exists across ethnicities and individuals [13].

The defining structural unit of WAT is the unilocular adipocyte, which is a large spherical cell that contains a single lipid droplet occupying most of the intracellular space. The cytoplasm is thinly distributed, and mitochondria are relatively few, distinguishing white adipocytes morphologically and functionally from their brown and beige counterparts [9,12]. These adaptations optimise white adipocytes for long-term energy storage in the form of triglycerides.

Functionally, WAT is far more than a passive reservoir of lipids. It plays central roles in systemic metabolic regulation, including endocrine signalling via adipokines (such as leptin and adiponectin), energy homeostasis, thermoregulation, immune responses, and glucose metabolism [15,16]. However, an imbalance in these functions can promote pathological processes. When energy intake chronically exceeds expenditure, WAT expands via hypertrophy and/or hyperplasia, often triggering inflammatory cascades, extracellular matrix remodelling, hypoxia, and fibrosis [17,18]. These maladaptive changes contribute to insulin resistance, dyslipidaemia, hypertension, and atherosclerosis, which characterise the obesity-associated metabolic syndrome [12,15,19,20].

### 1.2. Beige Adipose Tissue

Beige adipocytes originate from a precursor population resembling vascular smooth muscle-like cells that are negative for the transcription factors paired box 7 (PAX7) and myogenic factor 5 (MYF5), with PAX7 being essential for the maintenance of quiescent muscle satellite cells and MYF5 playing a crucial role in the early commitment of these progenitors to the myogenic lineage. In vivo lineage-tracing experiments using Myh11-Cre models have confirmed that at least a subset of beige cells arises from this smooth muscle-like lineage, rather than the myogenic line that gives rise to classical brown adipocytes [21].

Despite their different developmental origin, beige and brown adipocytes share a key transcriptional regulator, the PR domain containing 16 (PRDM16), which orchestrates thermogenic gene programmes. PRDM16 overexpression in subcutaneous WAT induces thermogenic genes such as UCP1, cell death-inducing DNA fragmentation factor-like effector A (CIDEA), and peroxisome proliferator-activated receptor gamma coactivator 1-α (PGC1α), enabling the conversion of white or beige progenitors into thermogenically active cells [21,22].

Morphologically, beige adipocytes are distinguishable by multilocular to unilocular lipid droplets and an intermediate mitochondrial density. They possess a moderate mitochondrial content, greater than WAT but lower than BAT [23]. Under thermoneutral conditions, they resemble white adipocytes, but upon exposure to cold, β-adrenergic agonists, peroxisome proliferator-activated receptor (PPARγ) agonists (for example, “rosiglitazone”), or exercise, BeigeAT undergoes “browning”, increasing mitochondrial biogenesis and upregulating thermogenic genes, including UCP1, CIDEA, and PGC1α [21,24].

Although BeigeAT can adopt a white-fat-like phenotype under warm ambient conditions, it maintains the potential to activate a thermogenic programme under appropriate stimuli. However, human epidemiological and imaging studies indicate that the abundance and functional relevance of beige fat in adult humans can be variable and context-dependent, warranting further investigation [25].

### 1.3. Brown Adipose Tissue

During human foetal development, classical BAT arises from a specialised subpopulation of mesodermal progenitor cells expressing PAX7, MYF5, and engrailed 1 (EN1), which also contributes to skeletal muscle and dermal lineages [22,26,27]. After birth, interscapular brown fat aids neonates in withstanding hypothermia through non-shivering thermogenesis [12]. However, BAT disappears as the body matures into adulthood [1,28].

In certain situations, such as cold exposure, research has shown that adults can reactivate brown fat, which accounts for only ~1.5% of body mass and 4.3% of total adipose tissue [12]. Adult BAT has been reported to be present in cervical, supraclavicular, mediastinal, paravertebral, axillary, abdominal, and perirenal localisations [29,30,31,32,33]. Furthermore, molecular imaging has increasingly demonstrated BAT activity in diverse clinical contexts, including oncological and endocrine pathologies [31,32,33].

**Figure 1:**
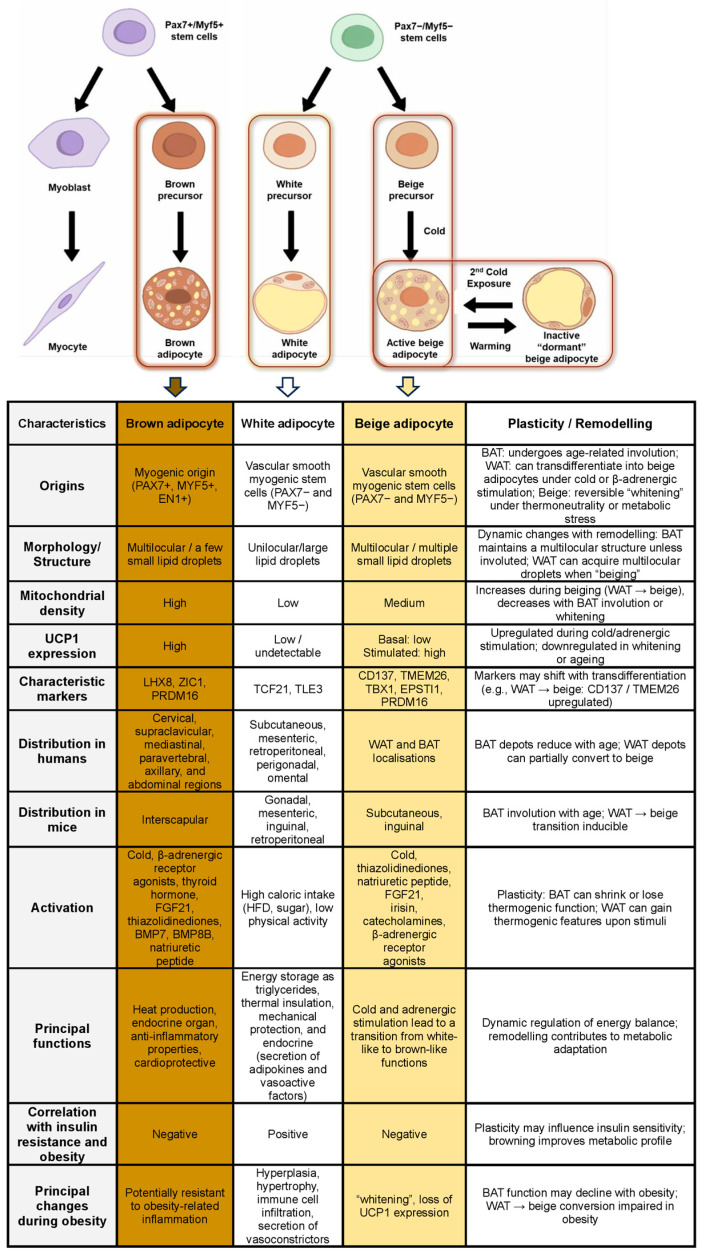
Overview of the origins and distinctive features of white, beige, and brown adipocytes [9,12,37,38].

Brown adipocytes are characterised by multiple small lipid droplets and a notably dense population of mitochondria, which imparts their distinctive brown nuance [12,34]. This multilocular architecture and substantial sympathetic nervous system (SNS) innervation facilitate rapid mobilisation of stored lipids for heat production. The UCP1 protein, localised within the mitochondrial inner membrane, disrupts the proton gradient generated during oxidative phosphorylation, releasing energy as heat rather than synthesising adenosine triphosphate (ATP). This mechanism underlies non-shivering thermogenesis, a key physiological function of BAT [35,36,37]. Furthermore, reviews highlight pathways for UCP1-independent thermogenesis in brown and beige adipocytes, such as creatine- and calcium-mediated systems, indicating a broader thermogenic capacity within mitochondrial networks [34,37] (Figure 1).

In addition to sympathetic stimulation, several endocrine and paracrine factors strongly modulate BAT development and thermogenic activation. Cardiac natriuretic peptides, such as atrial natriuretic peptide (ANP), have been shown to directly induce lipolysis and UCP1 expression in both human and murine adipocytes through cGMP–p38 MAPK signalling [39].

Thyroid hormone signalling is also indispensable, as triiodothyronine (T3) enhances mitochondrial biogenesis and UCP1 transcription in brown adipocytes, and local activation by type II deiodinase is critical for full thermogenic competence [40,41].

Bone morphogenetic protein 7 (BMP7) promotes commitment of mesenchymal progenitors toward the brown adipocyte lineage and potently induces UCP1 expression and mitochondrial biogenesis in vitro and in vivo [42].

Furthermore, the myokine irisin, released from skeletal muscle during exercise, was shown to drive browning of white adipose tissue and increase UCP1 levels through the induction of thermogenic gene programmes [43].

Collectively, these findings reinforce the integrative role of endocrine and paracrine cues in the regulation of BAT and its systemic metabolic effects.

## 2. How Does BAT Work?

BAT primarily functions to prevent hypothermia by releasing excess energy as heat, and notably, thermogenesis can occur without triggering muscle shivering in response to cold exposure, thus helping maintain thermal and energy balance [44].

Beyond heat production, both BAT and beige fat play essential roles in metabolic regulation by clearing various substrates from circulation, including glucose, fatty acids, cholesterol, and branched-chain amino acids (BCAAs) [45,46,47]. Significantly, BAT exhibits higher expression of BCAA catabolic enzymes, such as branched-chain amino acid aminotransferase 1 or 2 (BCAT1/2) and branched-chain keto acid dehydrogenase e1 subunit β (BCKDHB), compared to subcutaneous white fat. PET imaging studies using F-18 fluciclovine show remarkably greater BCAA uptake in supraclavicular BAT depots, which is diminished in conditions like obesity and type 2 diabetes [45].

At the tissue level, thermogenic fat is distinguished by lower levels of inflammation and fibrosis, alongside increased angiogenesis and lipid turnover, collectively contributing to tissue integrity and systemic metabolic homeostasis [44].

Furthermore, BAT and beige adipocytes secrete signalling molecules, termed batokines, that act through autocrine, paracrine, and endocrine pathways. These include hormones (e.g., fibroblast growth factor 21 (FGF21), neuregulin 4 (NRG4), interleukin 6 (IL-6), vascular endothelial growth factor A (VEGF-A)), lipid mediators (e.g., 12,13-dihydroxy-9Z-octadecenoic acid (12,13-diHOME)), and exosomal microRNAs (e.g., microRNA-99b (miR-99b)). Collectively, batokines enhance insulin sensitivity, regulate lipid and glucose metabolism, and influence vascular and immune functions. Beyond thermogenesis, BAT also contributes to systemic lipid clearance and protection against atherosclerosis, underscoring its role as a regulator of metabolic and cardiovascular health [32,33,48,49].

**Figure 2:**
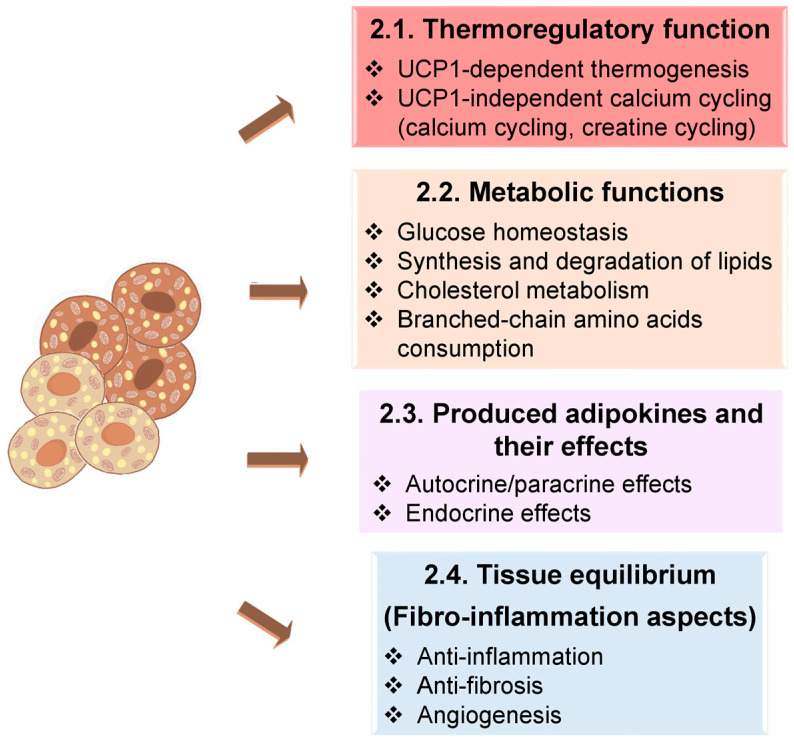
Principal functions of brown and beige adipocytes [50].

These combined actions of BAT and beige fat, thermogenesis, substrate clearance, tissue remodelling, and adipokines secretion, emphasise their potential as therapeutic targets in combating obesity, type 2 diabetes, and related metabolic disorders. These functions are summarised in Figure 2, which serves both as a schematic overview and as a structural guide to the discussion that follows [49,50].

### 2.1. Thermoregulatory Function

Since the discovery of brown fat in human adults, there has been a growing interest in understanding its physiology and function. Preserving the stability of the body’s internal temperature is essential for appropriate physiological functioning. Known as a non-shivering “heat producer”, BAT mainly works to sustain an optimal body temperature [50]. This primary function is predominantly mediated by its abundance of UCP1+ mitochondria, along with numerous intracellular triglyceride droplets [9].

Beyond the primary role of BAT mitochondria in synthesising ATP, they are also capable of generating heat. During ATP production, the F1F0-ATPase complex in the mitochondrial inner membrane depends on an electrochemical gradient formed by the translocation of H^+^ ions. This proton gradient is maintained by the tricarboxylic acid (TCA) cycle and the electron transport chain (ETC), which comprises complexes I–IV (with complex II formed by the TCA enzyme succinate dehydrogenase), coenzyme Q (CoQ), and cytochrome C (Cyt C) [1,51]. Upon β-adrenergic stimulation, UCP1, present in the inner mitochondrial membrane of brown and beige adipocytes, becomes activated. This functional anion/H^+^ symporter dissipates the proton gradient by shuttling H^+^ back into the mitochondrial matrix, uncoupling substrate oxidation from ATP production [50,52]. As a consequence, ATP synthesis is inhibited, and the released energy is dissipated as heat. The rate of heat generation in active BAT can reach up to 300 W/kg, vastly surpassing the 1 W/kg typical of other tissues [51,53].

Initially, it was believed that UCP1-mediated uncoupling required intracellular free fatty acids (FAs), released through local lipolysis in brown adipocytes. Cold exposure and β3-adrenergic signalling induce lipolysis, producing FAs that directly bind and activate UCP1, supporting β-oxidation and thermogenesis. Earlier studies using genetic ablation of adipose triglyceride lipase (ATGL) or its coactivator comparative gene identification-58 (CGI-58) suggested that local brown fat lipolysis might not be strictly necessary, as heat production in BAT was maintained under cold exposure [54,55]. However, more recent work has refined this view: In a BAT-specific ATGL/hormone-sensitive lipase (HSL) double knockout model, intracellular lipolysis was shown to be critical for thermogenic competence during cold exposure under fasting conditions, with impaired oxidative activity and substrate utilisation detected [56]. Interestingly, this requirement appears alleviated in the fed state, supporting a context-dependent reliance on intracellular versus circulating fatty acids for BAT thermogenesis. After lipolysis in WAT, these FAs may still be transported to brown adipocytes via the cluster of differentiation 36 (CD36), which facilitates their uptake across the plasma membrane [57,58].

Moreover, hepatic breakdown of WAT-derived fatty acids promotes the synthesis of acylcarnitines, a class of FA derivatives that can be transported to BAT and utilised to enhance mitochondrial β-oxidation and heat generation, thus highlighting an inter-organ collaboration between WAT, the liver, and BAT [59]. Importantly, the oxidation of long-chain fatty acids within brown adipocytes requires their prior transport into the mitochondrial matrix via the carnitine shuttle system, a multi-step process involving carnitine palmitoyltransferase I (CPT1), carnitine-acylcarnitine translocase (CACT), and carnitine palmitoyltransferase II (CPT2). This mechanism is obligatory for enabling β-oxidation and sustaining UCP1-dependent thermogenesis [60,61].

In beige adipocytes, an additional thermogenic mechanism, superstoichiometric futile creatine cycling, has been identified. In this pathway, ATP produced by the ETC is transported from the mitochondrial matrix to the intermembrane space by the adenine nucleotide translocator (ANT). This ATP is used by creatine kinase B (CKB) to convert creatine into phosphocreatine (PCr). Subsequently, tissue-nonspecific alkaline phosphatase (TNAP) hydrolyses PCr back to creatine, generating heat as a byproduct of this futile cycle [1,51]. Moreover, in this context, the term superstoichiometric refers to the fact that each round of PCr hydrolysis consumes more ATP than would be expected from a single stoichiometric reaction, thereby amplifying ATP turnover and dissipating energy as heat. This property makes creatine cycling a highly efficient thermogenic pathway in beige adipocytes, complementing UCP1-independent mechanisms [62].

Studies suggest that futile creatine cycling operates independently of UCP1 and may contribute to thermogenesis under specific stimuli such as pharmacologic agents (e.g., β3-agonists) or exercise-induced browning of WAT. This pathway is gaining attention for its therapeutic potential in obesity and metabolic syndrome, especially in individuals with low UCP1 activity [63,64].

Researchers indicate that post-translational modifications can regulate mitochondrial uncoupled respiration. Upon cold exposure, reactive oxygen species (ROS) induce sulfenylation of cysteine residue Cys253 in UCP1, a site located near the purine nucleotide-binding interface within the inner mitochondrial membrane, thereby promoting thermogenic activity [65]. Furthermore, studies have demonstrated that this sulfenylation is essential for proper UCP1 function, as mutation of Cys253 leads to a significant reduction in norepinephrine-induced activity. Another post-translational modification influencing UCP1 is succinylation at two lysine residues in the mitochondrial matrix: Lys56 and Lys151. Substituting these lysines with glutamic acid results in protein instability and diminished UCP1 function. Importantly, mitochondrial desuccinylase sirtuin 5 (SIRT5) can reverse succinylation, thereby regulating BAT’s primary thermogenic protein activity [66].

Heat generation can also occur via UCP1-independent mechanisms such as calcium (Ca^2+^) cycling. Maintaining intracellular Ca^2+^ homeostasis involves multiple processes. Sarcoplasmic/endoplasmic reticulum calcium ATPase (SERCA) pumps Ca^2+^ into the sarcoplasmic/endoplasmic reticulum in muscle cells, while inositol trisphosphate receptors (IP3R) or ryanodine receptors (RYRs) mediate its release. Under specific conditions, characterised by a high ATP/ADP ratio and elevated cytosolic Ca^2+^ concentration, ATP hydrolysis by SERCA becomes uncoupled from Ca^2+^ transport. This uncoupling causes continuous ATP consumption and dissipates energy as heat. In skeletal muscle, this process is estimated to generate approximately 14–16 kcal/mol of Ca^2+^ transported, though exact values vary by tissue [67]. Notably, anaesthetics or mutations in RYR1 can cause uncontrolled Ca^2+^ release, leading to malignant hyperthermia.

Type 1 SERCA (SERCA1), specific to skeletal muscle, interacts with the micropeptide sarcolipin (Sln), which uncouples ATP hydrolysis from Ca^2+^ transport and promotes non-shivering thermogenesis [68]. Although most research on UCP1-independent thermogenesis focuses on skeletal muscle, BeigeAT expresses type 2b SERCA (SERCA2B) and can similarly generate heat via Ca^2+^ cycling [69]. Upon cold exposure, two adrenergic pathways are activated in beige fat:

❖ The import of calcium ions into the intracellular space is stimulated by activated α1-adrenergic receptors.
❖ Type 2 RYR (RYR2) is stimulated by activated β3-adrenergic receptors, resulting in an increase in calcium ion discharge from the endoplasmic reticulum.

**Figure 3:**
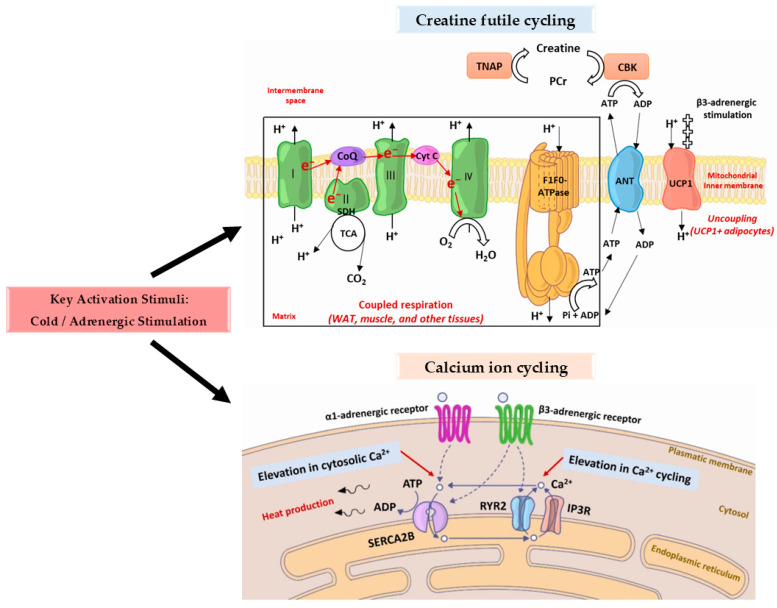
UCP1-independent thermogenic mechanisms in thermogenic adipose tissues [1,50,51].

Evidence has confirmed these mechanisms in vivo using an optogenetic approach to specifically induce UCP1-independent Ca^2+^ cycling-mediated thermogenesis, which effectively prevented diet-induced obesity by increasing whole-body energy expenditure [70]. Furthermore, it was shown that a high-carbohydrate, low-protein diet activates 5′ AMP-activated protein kinase (AMPK), which in turn stimulates SERCA activity and thermogenesis in subcutaneous white adipocytes [50,71] (Figure 3, Table 1).

In addition to calcium and creatine cycling, a futile lipid cycle has been proposed as another UCP1-independent thermogenic mechanism. This process involves continuous triglyceride hydrolysis and re-esterification, consuming ATP and dissipating energy as heat, and acts as an intra-adipocyte metabolic rheostat [77]. Experimental data demonstrated that in UCP1-knockout brown adipocytes, futile lipid cycling is recruited during cold exposure and significantly contributes to heat production and total energy expenditure [78].

Alongside these mechanisms, recent work has identified Chromosome 4 open reading frame 3 (C4orf3) (also termed ALN) as an endoplasmic reticulum-anchored peptide that acts as a molecular resistor of SERCA2b-mediated Ca^2+^ import. By uncoupling ATP hydrolysis from Ca^2+^ transport, C4orf3 converts SERCA2B activity into an exothermic process, thereby promoting UCP1-independent thermogenesis. Loss of C4orf3 markedly improves the energetic efficiency of SERCA2B but reduces heat generation and increases adiposity. Strikingly, combined ablation of C4orf3 and UCP1 severely compromises cold adaptation and systemic energy homeostasis [79].

### 2.2. Principal Metabolic Functions

While the thermogenic function of BAT has long been recognised, research has increasingly highlighted its broader metabolic significance, particularly its potential role in maintaining systemic energy homeostasis and mitigating a range of metabolic disorders [80].

#### 2.2.1. Glucose Homeostasis

BAT fulfils its thermogenic role by actively absorbing glucose following catecholamine stimulation. In parallel, insulin acts synergistically by binding to insulin receptors on brown adipocytes and activating downstream signalling, which drives the translocation of glucose transporter type 4 (GLUT4) to the cell surface. This mechanism significantly enhances the movement of glucose into the cells, thereby promoting intracellular glucose uptake [50]. This metabolic activity contributes to systemic glycaemic regulation. Importantly, human meta-analyses confirm that cold-induced BAT activation significantly elevates circulating FAs and lipid mediators, facets of systemic metabolic signalling without necessarily altering fasting insulin or glucose levels [80].

Moreover, BAT mass and activity decline with age and in metabolic disorders, a process often referred to as BAT involution. This reduction in functional brown adipocytes may contribute to impaired glucose uptake, diminished insulin sensitivity, and systemic metabolic dysregulation [81,82]. Animal studies suggest that age-related downregulation of insulin receptor expression and reduced adrenergic responsiveness in brown adipocytes contribute to this decline, highlighting the physiological relevance of maintaining BAT function throughout life [83].

Of note, brown and white adipose tissues exhibit distinct glucose uptake mechanisms. While glucose entry into WAT is strictly dependent on insulin-stimulated GLUT4, brown adipocytes are capable of utilising glucose transporter type 1 (GLUT1) independently of insulin via adrenergic stimulation [84]. This pathway appears to involve direct regulation by the mechanistic target of rapamycin complex 2 (mTORC2), as demonstrated in murine models [85].

As previously discussed, adipocytes expressing UCP1 play a crucial role in maintaining glucose homeostasis. This function has been substantiated by numerous studies:

❖ Genetic deletion of UCP1 in murine models leads to hyperglycaemia, hyperinsulinaemia, and increased adiposity [86].
❖ Brown fat transplantation enhances insulin sensitivity and glucose uptake in both WAT and BAT through insulin-mediated pathways [87]. Similarly, β3-adrenergic stimulation using CL316243 confirmed improved glucose handling without significant changes in body mass [88].
❖ Overexpression of PRDM16, a key transcriptional regulator of beige adipocyte identity, leads to elevated BeigeAT activity and improved insulin and glucose responsiveness. Conversely, PRDM16-deficient mice show diminished beige fat development and impaired insulin sensitivity [22,89].
❖ In models with PRDM16 overexpression but lacking UCP1, glucose uptake remained elevated, suggesting that BeigeAT retains insulin sensitivity independently of UCP1-mediated thermogenesis [69].
❖ Studies have identified a non-thermogenic function of BAT in the regulation of systemic insulin sensitivity. Specifically, impaired mitochondrial BCAA uptake via the transporter Solute Carrier Family 25 Member 44 (SLC25A44) in BAT leads to intracellular accumulation of BCAAs and their ketoacid derivatives (BCKAs), resulting in elevated oxidative stress, disrupted hepatic insulin signalling, and widespread insulin resistance. Notably, these effects occur independently of changes in energy expenditure or overall adiposity [90,91].
❖ Batokine production, including FGF21, appears to connect BAT to systemic glucose metabolism. FGF21 released by activated BAT exerts endocrine actions on liver and adipose tissue, enhancing glucose disposal and promoting WAT browning [92,93,94,95,96].
❖ In obese individuals, reduced BAT thermogenesis and insulin sensitivity are commonly observed. Nonetheless, multiple studies suggest that activation of thermogenic pathways, regardless of the stimulus, can restore glycaemic control and insulin responsiveness in this population [97]. Indeed, human imaging studies suggest that declines in BAT glucose uptake often precede measurable changes in thermogenic activity and may serve as sensitive early indicators of metabolic dysfunction [31,32,98].

Beyond adipose-specific effects, both BAT and BeigeAT are increasingly recognised as regulators of whole-body glucose metabolism, with potential roles in modulating insulin action in tissues such as the liver and skeletal muscle. Although these systemic interactions remain incompletely characterised, current evidence points to their involvement in energy expenditure, anti-inflammatory signalling, and endocrine crosstalk with other metabolic organs [50].

Recent studies have highlighted an additional metabolic aspect of BAT: its potential to suppress tumour growth by outcompeting cancer cells for glucose. Exposure of tumour-bearing mice to cold conditions activates BAT, leading to a substantial decrease in blood glucose levels and reducing glycolysis in cancer cells. Furthermore, implanting engineered adipocytes that utilise increased amounts of glucose and fatty acids has been shown to suppress tumour progression, further supporting a direct competition for metabolic resources between BAT and cancer cells [99,100]. These findings underscore the multifaceted effects of BAT in metabolic regulation and its possible implications in cancer biology.

#### 2.2.2. Synthesis and Degradation of Lipids

Free FAs are a vital energy source for BAT and BeigeAT, complementing glucose to sustain their thermogenic and metabolic functions [50,56]. Accordingly, brown and beige adipocytes are implicated in both lipid synthesis and catabolic pathways.

Preclinical studies have demonstrated that cold exposure in mice enhances fatty acid uptake by BAT, reducing circulating triglyceride-rich lipoproteins (TRLs) [101]. Under hyperlipidaemic conditions, BAT activation, whether via cold stimulation or administration of the β3-adrenergic agonist CL316243, facilitates TRL clearance through apolipoprotein A-V (APOA5)-dependent pathways. This process is critically mediated by lipoprotein lipase (LPL) and the fatty acid translocase CD36, ultimately leading to reduced plasma lipid levels and conferring protection against atherosclerosis in hyperlipidaemic models [47].

Moreover, BAT activation has been associated with increased high-density lipoprotein cholesterol (HDL-C), enhancing cholesterol efflux and HDL regeneration [101]. In humans, elevated BAT volume correlates with lower triglyceride levels and faster clearance of very-low-density lipoprotein triglycerides (VLDL-TG) and free FAs, without affecting their secretion rates [102]. A large human cohort study similarly reported that active BAT is linked to lower plasma triglycerides, higher HDL-C, and a favourable total cholesterol (TC)/HDL-C ratio [103]. Another recent PET/CT-based analysis confirmed that BAT-positive individuals exhibit significantly higher HDL-C and lower low-density lipoprotein cholesterol (LDL-C)/HDL-C ratios [104].

Lipidomic analyses have revealed that BAT undergoes dynamic remodelling during metabolic challenge, modulating its lipid mediator profile and systemic impacts [105]. Mechanistic reviews further underscore that, beyond thermogenesis, BAT may also contribute to systemic lipid metabolism through cross-tissue signalling pathways that influence lipid equilibrium [106].

A growing body of research has shed light on the influence of both BAT and BeigeAT on lipid metabolism, particularly through their roles in fat-driven inter-organ communication. This complex, multi-organ crosstalk is typically initiated by cold exposure and unfolds through a series of tightly regulated steps, ultimately contributing to the restoration of systemic lipid homeostasis [50,107,108]:

❖ Cold stimulation of the body initiates hepatic conversion of cholesterol into bile acids, followed by their excretion.
❖ These bile acids play a key role in maintaining intestinal microbiota homeostasis.
❖ In turn, the bile acids stimulate gut microbial activity and metabolite production.
❖ The combination of microbiota-derived signalling molecules and bile acids in the circulation activates brown adipocytes.
❖ Activated BAT secretes phospholipid transfer protein (PLTP).
❖ PLTP supports HDL biogenesis, particularly in structural stabilisation.
❖ The resulting HDL particles reduce circulating ceramide and phospholipid levels and enhance lipid excretion via faeces.

In contrast to these protective mechanisms, statin therapy has been shown to impair BAT and BeigeAT function by interfering with the mevalonate and geranylgeranyl pyrophosphate biosynthesis pathways, potentially limiting their lipid-processing capacity [109].

Taken together, these findings illustrate the complex and systemic role of brown and beige adipose tissues in lipid synthesis and clearance. Nonetheless, further clinical and mechanistic research is required to fully characterise these endocrine and metabolic interactions.

#### 2.2.3. Branched-Chain Amino Acid Consumption

Extensive research has characterised BCAAs, leucine (Leu), valine (Val), and isoleucine (Ile) as essential not only for protein synthesis, athletic recovery, and cognitive and psychomotor performance [110,111], but also for their linkage with BAT and BeigeAT activity [90,112]. Cold exposure in humans with functional BAT/BeigeAT has been shown to reduce circulating BCAA levels, reflecting increased metabolic clearance [50,90].

In murine models, impaired BCAA catabolism, resulting from deficient mitochondrial enzymes such as branched-chain keto acid dehydrogenase e1 α subunit (BCKDHA) or branched-chain amino acid transaminase 2 (BCAT2), leads to elevated plasma BCAAs, reduced insulin sensitivity, glucose intolerance, and weight gain [90,113,114]. Dysregulated BCAA metabolism is now widely recognised as a hallmark of obesity-related insulin resistance [113,115].

BAT and BeigeAT play a critical role in systemic BCAA clearance. Cold-activated BAT significantly increases BCAA-nitrogen flux and metabolite synthesis [91], while human supraclavicular BAT demonstrates higher uptake of a Leu analogue (^18^F-fluciclovine) and elevated expression of BCAA catabolic genes in lean individuals compared to those with obesity or T2D [45].

In addition, BCAAs serve as substrates for the generation of monomethyl branched-chain fatty acids (mmBCFAs) in BAT and other tissues, especially following cold exposure. These unique lipids derive from BCAA carbon and contribute to adipose tissue lipid synthesis [114,115]. Obese individuals exhibit reduced blood mmBCFA levels, whereas weight-loss interventions, including bariatric surgery, restore their circulating concentrations [114]. Intriguingly, mmBCFAs appear to enhance skeletal muscle insulin sensitivity when elevated in BAT/BeigeAT [116]. Although the complete mechanistic pathways of mmBCFAs remain under investigation, it is apparent that BAT-mediated BCAA consumption and mmBCFA generation significantly contribute to systemic glycaemic and insulin regulation.

### 2.3. Produced Adipokines and Their Effects

Brown and beige fats are metabolically active tissues that secrete a complex array of signalling proteins, often referred to as adipokines, adipocytokines, or, more specifically, batokines. These bioactive molecules function in both autocrine/paracrine and endocrine manners and play a pivotal role in maintaining whole-body metabolic homeostasis [1,9]. Furthermore, experimental studies have shown that the transplantation of a minimal amount of BAT is sufficient to exert systemic metabolic benefits, influencing glucose metabolism, insulin sensitivity, and energy expenditure across multiple organs [87,117].

Support for these endocrine effects has been established through comparisons of animal models either lacking BAT or genetically modified to lack the mitochondrial UCP1. In both scenarios, substantial physiological alterations were observed, indicating that thermogenic fat plays more than a local metabolic role [86,118]. Additionally, evidence shows that PRDM16-driven beige adipocytes are capable of cytokine secretion even in the absence of UCP1, further demonstrating that PRDM16 acts as a master regulator of adipose tissue endocrine activity [69]. This finding suggests that beige adipose tissue may retain key metabolic functions independently of thermogenesis, thus expanding our understanding of its role in systemic homeostasis.

Expanding on this concept, researchers have catalogued numerous BAT/BeigeAT-derived polypeptides that impact not only neighbouring cells but also distant tissues via systemic circulation [107,119,120,121]. These secreted molecules can be broadly divided into two classes: those acting locally (autocrine/paracrine) and those with endocrine potential, such as FGF21, NRG4, and IL-6, which contribute to interorgan communication (Table 2).

In addition to peptide-based signals, BAT also produces specific lipid-derived metabolites, including 12,13-diHOME. Exposure to cold has been shown to elevate circulating levels of this oxylipin, which promotes lipid uptake in brown adipocytes [61]. Furthermore, physical activity can stimulate BAT to release 12,13-diHOME, enhancing fatty acid oxidation in skeletal muscle, thus linking thermogenic fat to exercise-induced metabolic adaptations [131].

Moreover, brown fat is capable of generating extracellular vesicles carrying functional microRNAs, which participate in both local and systemic gene regulation. These vesicle-bound microRNAs mediate communication with other tissues, such as the liver, pancreas, and skeletal muscle, and may play a role in modulating insulin signalling and lipid metabolism [132,133].

**Figure 4:**
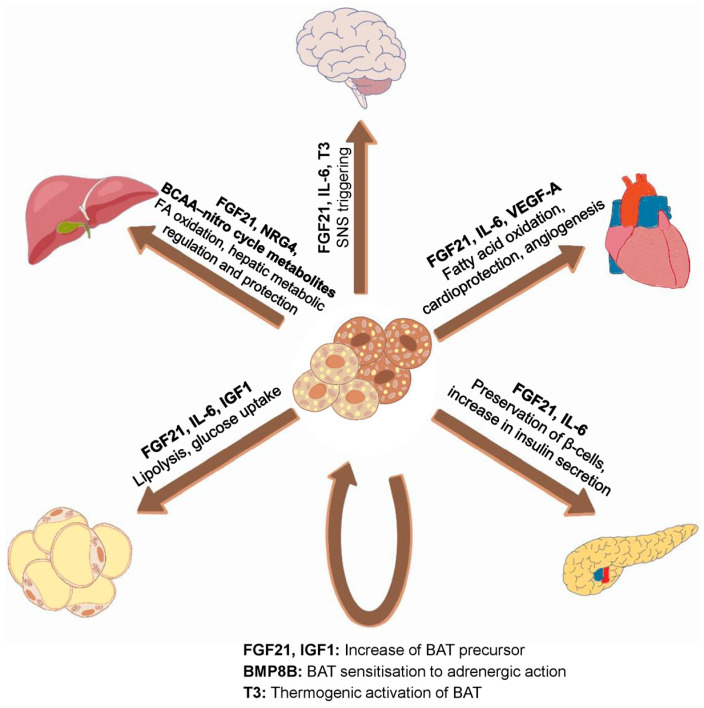
Brown/Beige adipocytes: Part of a multi-organ crosstalk [33,37,48,134].

Despite this growing body of knowledge, the complete spectrum of batokines, particularly those with therapeutic relevance, remains incompletely elucidated. It is therefore essential to identify new molecular actors secreted by BAT/BeigeAT and to characterise their target tissues, mechanisms of action, and potential as clinical tools for treating obesity-related metabolic disorders (Figure 4).

### 2.4. Fibro-Inflammatory Aspects

The structural integrity and intercellular signalling of adipocytes are heavily reliant on the ECM and its intricate composition [5,135]. This matrix is composed of structural proteins, including collagens (types I, III, and VI), elastin, and fibronectin, as well as integrin receptors, growth factors such as transforming growth factor-β (TGF-β) and connective tissue growth factor (CTGF), and remodelling enzymes like matrix metalloproteinases (MMP2, MMP9, and MMP14), along with intracellular peptidase D [5,136,137]. As mentioned above, the constitution and dynamic remodelling capacity of this matrix directly influence adipose tissue expansion, differentiation, and overall functionality [138].

An imbalanced ECM, particularly an excess of pro-fibrotic components like collagen types I, III, and VI, can result in dysfunctional, fibrotic adipose tissue [139]. This maladaptive remodelling is commonly found in obese individuals with associated metabolic comorbidities, characterised by chronic low-grade inflammation and increased infiltration of pro-inflammatory immune cells [17]. A strong correlation has been established between obesity-driven ECM dysregulation in WAT, insulin resistance, and increased susceptibility to T2D [140,141,142]. Supporting this, studies using genetically modified mice deficient in collagen VI demonstrated improved insulin sensitivity, reduced white fat fibrosis, and lower T2D risk [143].

In contrast, brown and beige fats exhibit protective anti-fibrotic properties. These benefits were evident in preclinical models exposed to cold or PRDM16 stimulation, both of which promoted ECM remodelling, favouring adipocyte health [144,145]. Interestingly, UCP1-deficient animal models also demonstrated resistance to adipose tissue fibrosis, suggesting that thermogenic protection may be partially independent of UCP1 expression [144].

Two principal molecular pathways have been elucidated by which thermogenic brown and beige adipose tissues exert anti-fibrotic effects. First, PRDM16 stimulates the local secretion of β-hydroxybutyrate (BHB), which suppresses fibrogenesis in adipose precursor cells and promotes beige adipocyte differentiation [145]. Second, upon cold exposure, PRDM16 forms a transcriptional complex with the cold-inducible factor general transcription factor 2 I repeat domain-containing 1 (GTF2IRD1), which represses TGF-β-driven pro-fibrotic gene expression. Notably, these mechanisms function independently of UCP1 activity and play a critical role in ECM remodelling and adipocyte health maintenance [144,145].

In addition, the benefits of BAT/BeigeAT in controlling blood glucose levels have been shown to be related to their capacity to fight fibro-inflammation. These findings have been demonstrated in a group of animals that showed an enhancement in insulin and glucose tolerances after receiving BHB. Moreover, the high expression of GTF2IRD1 in thermogenic fat has also been correlated with better glucose and insulin metabolism [144].

It has been observed that the composition of the ECM is closely linked to the thermogenic activity of brown and beige adipose tissues, particularly their capacity to generate heat [121,146]. Studies investigating ECM remodelling in BAT have reported heterogeneous findings. In drug-induced thermogenesis, certain ECM components, such as hyaluronan and laminin α 4 (LAMA4), were shown to decrease [147], whereas Porras et al. [148] did not involve such pharmacological intervention. Nevertheless, in fibrotic states, excessive ECM deposition is consistently associated with impaired BAT function [138]. For example, fibro-inflammatory processes and metabolic disturbances associated with brown fat have been linked to elevated levels of endotrophin (ETP), a profibrotic mediator derived from the cleavage of the collagen type VI α 3 (COL6A3) chain [149]. Further experimental studies have identified that inhibition of VEGF results in type I collagen accumulation, increased macrophage infiltration, and the degeneration of BAT cells [150].

Another important aspect is that both white and brown fat contribute significantly to immune system regulation by modulating immune responses through the secretion of tissue-specific signalling molecules that influence the complement cascade [120]. Notably, the complement factor H (CFH) was primarily identified in the BAT secretome. CFH is a negative regulator of the innate immune system and inhibits the alternative pathway of the complement system. This finding aligns with the increased anti-inflammatory capacity of brown adipocytes compared to white fat cells [120].

In individuals with obesity, white adipocytes produce a range of pro-inflammatory cytokines, including tumour necrosis factor-α (TNF-α), interferon-γ, interleukin-1β (IL-1β), and IL-6, which collectively increase macrophage infiltration in adipose tissue from below 10% to nearly 40% [151,152,153]. In parallel, early in the development of obesity, both CD4+ and CD8+ T cells become activated and proliferate within adipose depots, contributing to the initiation and maintenance of inflammation [154,155]. The resultant immune activation, combined with reduced insulin sensitivity in adipocytes, promotes excessive release of free FAs, which in turn stimulate hepatic gluconeogenesis, ultimately contributing to hyperglycaemia and the progression of type 2 diabetes [156].

**Figure 5:**
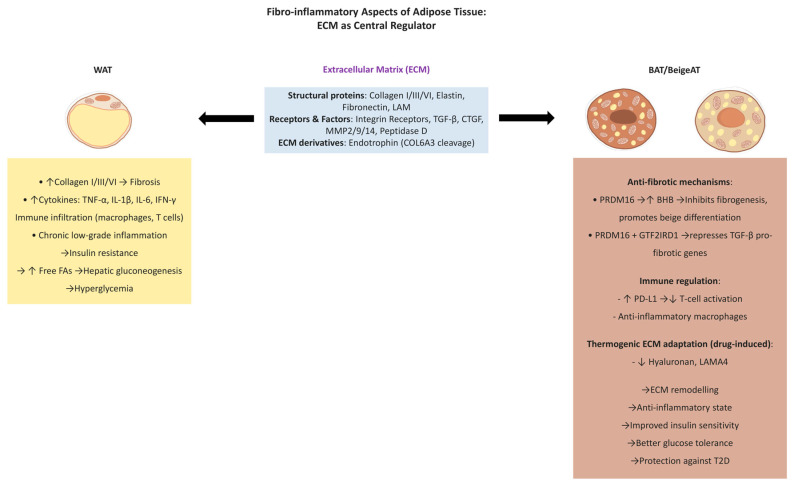
Fibro-inflammatory aspects of adipose tissue [1,5,17,120,121,135,136,137,138,139,140,141,142,143,144,145,146,147,148,149,150,151,152,153,154,155,156,157,158]. ↑ indicates increase, ↓ indicates decrease, → indicates leads to/causal relationship.

In contrast, BAT displays anti-inflammatory properties and may attenuate immune activation in obesity. For instance, research has shown that elevated expression of programmed death ligand 1 (PD-L1) in brown adipocytes reduces T-cell activation [157]. This upregulation of PD-L1 is also associated with improved insulin sensitivity [1]. Insights confirm that PD-L1 expression in BAT correlates with immune regulation and insulin tolerance in humans and is notably higher than in WAT [158] (Figure 5).

## 3. Conclusions

BAT has evolved from a physiological curiosity to a central player in metabolic homeostasis, immunomodulation, and inter-organ communication. Its unique origin, structural specialisation, and bioenergetic capacity, anchored in both UCP1-dependent and alternative thermogenic mechanisms, grant BAT and beige fat a distinct identity within the adipose lineage. More than a site of heat production, BAT/BeigeAT acts as a systemic metabolic regulator, capable of clearing glucose, lipids, and amino acids while secreting a diverse array of bioactive molecules that affect distant tissues.

The endocrine and paracrine networks emerging from thermogenic fat depots underscore their integration within broader physiological systems, including the liver, pancreas, skeletal muscle, cardiovascular system, and immune landscape. Furthermore, the anti-fibrotic and anti-inflammatory properties of BAT/BeigeAT not only sustain tissue plasticity but also offer protection against metabolic dysfunction and organ crosstalk failure.

As the global burden of obesity and metabolic syndrome escalates, BAT/BeigeAT offers a compelling therapeutic target, one that combines energy expenditure, substrate clearance, endocrine signalling, and tissue remodelling in a single physiological unit. Yet, despite significant progress in imaging, mechanistic understanding, and functional characterisation, the clinical translation of thermogenic fat-based strategies remains in its infancy.

The question is no longer whether brown and beige adipose tissues are relevant to adult human health, but how we can activate, sustain, and harness their full potential in real-world metabolic medicine. Future research must move beyond description and into intervention. Large-scale clinical trials, longitudinal metabolic imaging, and precise molecular targeting are urgently needed to bridge the gap between BAT/BeigeAT biology and therapeutic innovation. The next frontier in adipose research is not in understanding if thermogenic fat can be therapeutic, but how fast we can make that a reality.

## Figures and Tables

**Table 1:** Comparative mechanisms of UCP1-dependent and UCP1-independent thermogenesis in adipose tissues.

	UCP1-Dependent Thermogenesis	UCP1-Independent Thermogenesis
**Main Protein**	❖ UCP1	❖ SERCA, Sln, futile creatine cycling proteins
**Location**	❖ Inner mitochondrial membrane of brown and beige adipocytes	❖ Skeletal muscle, beige adipocytes, subcutaneous white adipocytes
**Activation Stimuli**	❖ Cold exposure, β3-adrenergic receptor stimulation, lipolysis-derived free FAs activate UCP1	❖ Cold exposure, β3-adrenergic receptor stimulation, α1-adrenergic receptor stimulation, exercise, pharmacological agents (β3-agonists), dietary cues (high-carbohydrate, low-protein)
**Energy Source**	❖ Proton gradient dissipation via H^+^ leak	❖ ATP hydrolysis uncoupled from Ca^2+^ transport (SERCA–Sln), creatine-phosphate futile cycling, Ca^2+^ cycling via RYR and IP3R
**Inhibitory Mechanisms**	❖ Purine nucleotides (ATP, guanosine diphosphate (GDP)) inhibit UCP1 activity	❖ Not applicable directly to a single uncoupler; regulation through protein signalling
**Post-translational Modifications**	❖ Sulfenylation of Cys253, succinylation at Lys56 and Lys151 modulated by SIRT5	❖ Not directly applicable, regulation mainly via protein–protein interactions and AMPK activation
**Role of ROS**	❖ Required for sulfenylation and activation of UCP1	❖ Indirect; ROS can influence Ca^2+^ signalling pathways
**Heat Production Efficiency**	❖ Up to 300 W/kg, highly efficient in brown adipocytes	❖ Lower than the UCP1 pathway per cell, but significant in muscle and beige fat; cumulative effect substantial
**Fatty Acid Requirement**	❖ Essential in the fasted state via ATGL/HSL-mediated intracellular lipolysis; dispensable in the fed state when exogenous substrates are available	❖ Independent of fatty acid oxidation; dependent on ATP hydrolysis
**Metabolic Benefits and Non-thermogenic Roles**	❖ Enhances glucose and insulin tolerance, protects against obesity and T2D, modulates ECM remodelling and immune profile	❖ Similar metabolic benefits, especially in individuals with low UCP1 activity; potential therapeutic target for obesity and metabolic syndrome; may impact fibro-inflammatory balance and metabolic resilience
**Additional Notes**	❖ UCP1 activity can be inhibited by mutations or post-translational modifications; requires an intact mitochondrial proton gradient	❖ Creatine futile cycling and Ca^2+^ cycling provide alternative thermogenic pathways; optogenetic studies confirm efficacy in vivo; regulated by AMPK and adrenergic receptors
**Key References**	❖ Chen et al., 2020 [51]; Cohen and Kajimura, 2021 [50]; Klingenberg, 2017 [52]; Jones et al., 2025 [72]; Bast-Habersbrunner and Fromme, 2020 [73]; Divakaruni et al., 2012 [74]; Mouisel et al., 2025 [56]	❖ Bunk et al., 2025 [63]; Kazak et al., 2015 [75]; Guarnieri et al., 2022 [76]; Vargas-Castillo et al., 2024 [64]

**Table 2:** Principal BAT/BeigeAT-derived polypeptides and their principal effects [48,50,66,122,123,124,125,126,127,128,129,130].

	Polypeptides	Effects/Organs
**Autocrine/Paracrine actions**	❖ BMP8B ❖ SLIT2 ❖ IL-6 ❖ FGF21	❖ Thermogenesis regulation
	❖ VEGF-A	❖ Vascularisation regulation
	❖ CXCL14 ❖ GDF15	❖ Immunity regulation
	❖ NGF1 ❖ S100B	❖ Fatty tissue innervation
**Endocrine actions**	❖ NRG4	❖ Hepatic lipogenesis inhibition
	❖ Myostatin/growth differentiation factor 8 (GDF8)	❖ Regulation of skeletal muscle function
	❖ FGF21	❖ Regulation of BeigeAT production and cardiac remodelling
	❖ PLTP	❖ Regulation of hepatic glucose and metabolism

## Data Availability

Not applicable.

## Abbreviations

The following abbreviations are used in this manuscript:

12,13-diHOME	12,13-dihydroxy-9z-octadecenoic acid
AMPK	AMP-activated protein kinase
ANP	atrial natriuretic peptide
ANT	adenine nucleotide translocator
APOA5	apolipoprotein A-V
ATGL	adipose triglyceride lipase
ATP	adenosine triphosphate
BAT	brown adipose tissue
BCAAs	branched-chain amino acids
BCAT1/2	branched-chain amino acid aminotransferase 1 or 2
BCAT2	branched-chain amino acid transaminase 2
BCKAs	branched-chain keto acids
BCKDHA	branched-chain keto acid dehydrogenase e1 α subunit
BCKDHB	branched-chain keto acid dehydrogenase e1 β subunit
BeigeAT	beige adipose tissue
BHB	β-hydroxybutyrate
BMP	bone morphogenetic protein
BMP7	bone morphogenetic protein 7
BMP8B	bone morphogenetic protein 8b
C4orf3	chromosome 4 open reading frame 3
CACT	carnitine-acylcarnitine translocase
Ca^2+^	calcium
CD36	cluster of differentiation 36
CFH	complement factor H
CGI-58	coactivator comparative gene identification 58
cGMP–p38 MAPK	cyclic guanosine monophosphate–p38 mitogen-activated protein kinase
CIDEA	Cell death–inducing DNA fragmentation factor-like effector A
CKB	creatine kinase B
COL6A3	collagen type VI α 3
CoQ	coenzyme Q
CPT1	carnitine palmitoyltransferase I
CPT2	carnitine palmitoyltransferase II
CTGF	connective tissue growth factor
CXCL14	C-X-C motif chemokine ligand 14
Cyt C	cytochrome C
ECM	extracellular matrix
EN1	engrailed 1
EPSTI1	epithelial stromal interaction 1
ETC	electron transport chain
ETP	endotrophin
FAs	fatty acids
FGF21	fibroblast growth factor 21
GDF15	growth differentiation factor 15
GLUT1	glucose transporter type 1
GLUT4	glucose transporter type 4
GTF2IRD1	general transcription factor 2 I repeat domain-containing 1
HDL-C	high-density lipoprotein cholesterol
IL-1β	interleukin-1β
IL-6	Interleukin-6
Ile	isoleucine
IP3R	inositol trisphosphate receptors
LAMA4	laminin α 4
Leu	leucine
LHX8	lim homeobox protein 8
LPL	lipoprotein lipase
miR-99b	microRNA-99b
mmBCFAs	monomethyl branched-chain fatty acids
MMP	metalloproteinase
mTORC2	mechanistic target of rapamycin complex 2
MYF5	myogenic factor 5
NGF	nerve growth factor
NRG4	neuregulin 4
PAX7	paired box 7
PCr	phosphocreatine
PD-L1	programmed death ligand 1
PGC1α	peroxisome proliferator-activated receptor gamma coactivator 1-α
Pi	inorganic phosphate
PLTP	phospholipid transfer protein
PPARγ	peroxisome proliferator activated receptor
PRDM16	pr domain containing 16
ROS	reactive oxygen species
RYR2	type 2 RYR
RYRs	ryanodine receptors
S100B	s100 calcium-binding protein B
SDH	succinate dehydrogenase
SERCA	sarcoplasmic/endoplasmic reticulum calcium ATPase
SERCA1	type 1 SERCA
SERCA2B	type 2b SERCA
SIRT5	desuccinylase sirtuin 5
SLC25A44	solute carrier family 25 member 44
SLIT2	slit guidance ligand 2
Sln	sarcolipin
SNS	sympathetic nervous system
T2D	type 2 diabetes
T3	triiodothyronine
TBX1	t-box 1
TCA	tricarboxylic acid
TCF21	transcription factor 21
TGF-β	transforming growth factor-β
TLE3	transducin-like enhancer of split 3
TMEM26	transmembrane protein 26
TNAP	tissue-nonspecific alkaline phosphatase
TNF-α	tumour necrosis factor-α
TRLs	triglyceride-rich lipoproteins
UCP1	uncoupling protein 1
Val	valine
VEGF-A	vascular endothelial growth factor A
VLDL-TG	very-low-density lipoprotein triglycerides
WAT	white adipose tissue
ZIC1	zinc finger protein of the cerebellum 1
